# Tip60 Phosphorylation at Ser 99 Is Essential for Autophagy Induction in *Bombyx mori*

**DOI:** 10.3390/ijms21186893

**Published:** 2020-09-20

**Authors:** Wenmei Wu, Kang Li, Haigang Zhao, Xianying Xu, Jing Xu, Man Luo, Yang Xiao, Ling Tian

**Affiliations:** 1 Guangdong Provincial Key Laboratory of Agro-Animal Genomics and Molecular Breeding/Guangdong Provincial Sericulture and Mulberry Engineering Research Center, College of Animal Science, South China Agricultural University, Guangzhou 510642, China; wuwenmei@stu.scau.edu.cn (W.W.); 20183139062@stu.scau.edu.cn (X.X.); zhibaoxujing@stu.scau.edu.cn (J.X.); luoman@stu.scau.edu.cn (M.L.); 2 Guangdong Province Key Laboratory for Biotechnology Drug Candidates, School of Life Sciences and Biopharmaceutics, Guangdong Pharmaceutical University, Guangzhou 510006, China; 3 Guangdong Provincial Key Laboratory of Insect Developmental Biology and Applied Technology, Institute of Insect Science and Technology, School of Life Sciences, South China Normal University, Guangzhou 510631, China; likang0118@foxmail.com (K.L.); hgzhao@sibs.ac.cn (H.Z.); 4 The Sericultural and Agri-Food Research Institute of the Guangdong Academy of Agricultural Sciences, Guangzhou 510610, China; xiaoyang@gdaas.cn

**Keywords:** BmTip60, autophagy, *Bombyx mori*, AMP-activated protein kinase, starvation

## Abstract

Tip60, a key histone acetyltransferase of the MYST family and member of the nuclear multimeric protein complex (NuA4), regulates the activity and stability of proteins involved in the cell cycle, DNA damage responses, autophagy, etc. However, the function and regulatory mechanism of Tip60 homolog in *Bombyx mori* are not elucidated. In the present study, *Bombyx* Tip60 (BmTip60) was functionally identified. Developmental profiles showed that the protein levels and nuclear localization of BmTip60 peaked in fat body during the larval–pupal metamorphosis when autophagy was intensive; simultaneously, the BmTip60 protein migrated to form an upper band as detected by Western blot. Interestingly, the upper band of BmTip60 was reduced by λ-phosphatase treatment, indicating that it was a phosphorylated form of BmTip60. Results showed that BmTip60 was promoted by starvation but not 20-hydroxyecdysone treatment. Transcription factor AMP-activated protein kinase (AMPK) affected by starvation was pivotal for BmTip60 protein migration. In addition, one mammalian phosphorylation site was identified in BmTip60 at Ser99, the constitutive-activation mutation of Ser99 to Asp99 but not its inactive mutation to Ala99 significantly upregulated autophagy, showing the critical role of phosphorylation at Ser99 for BmTip60-mediated autophagy. In conclusion, the starvation-AMPK axis promotes BmTip60 in *B. mori*, which was requisite for autophagy induction. These results reveal a regulatory mechanism of histone acetyltransferase Tip60 homologs by phosphorylation in insects, and sheds light on further related studies of acetylation regulation.

## 1. Introduction

Macroautophagy/autophagy is an evolutionarily conserved catabolic process to sequester intracellular components in the double-membrane autophagosomes, which is finally fused with lysosomes for bulk degradation of substrates [1]. Autophagy is crucial for maintaining cell homeostasis triggered by a wide variety of stresses, such as nutrient deprivation, organelle damage, oxidative stress, and protein aggregation [1,2]. Autophagosome maturation is a multiple-step process mainly including initiation, nucleation, elongation, and maturation, which depends on the participation of a series of autophagy-related (Atg) proteins [1,3]. ULK1/Atg1-Atg13 and BECN1/Atg6-PIK3C3/Vps34 (catalytic subunit of class III PtdIns3K) protein complexes are requisite for the initiation and nucleation of autophagosomes, respectively; autophagosome elongation and maturation are mediated by two ubiquitin-like conjugation systems Atg12–Atg5-Atg16L1 and Atg8–phosphatidyl ethanolamine (PE) [1,4].

Autophagy is strictly regulated by nutrients, energy, and insect molting hormone 20-hydroxyecdysone (20E) at transcriptional and post-translational levels [4,5,6,7,8]. Phosphorylation, acetylation, ubiquitination, SUMOylation, and methylation are well studied post-translation modifications (PTMs) of proteins, which play pivotal roles in biological processes in eukaryotes [9,10]. Histone acetyltransferases (HATs) and histone deacetylases (HDACs), which catalyze the acetylation and deacetylation of histone and nonhistone proteins, respectively, are associated with DNA repair, apoptosis, and angiogenesis in addition to autophagy [9,11,12,13]. In mammals, sirtuin 1 (SIRT1), histone deacetylase 6 (HDAC6), and HDAC1 deacetylate ATG proteins such as ATG5, ATG7, LC3/Atg8 promote autophagy [11,12,13]. Mammalian HDAC homologies such as Rpd3, HDAC1, HDAC3, HDAC6, and SIRT2 are suggested to link with stress resistance in *Drosophila melanogaster*, development in *Sarcophaga bullata*, and phase polymorphism in locusts [14,15,16]. Moreover, parasitoids are more likely to suppress host immune systems and development by impeding histone acetylation and deacetylation [17]. A recent study reveals that cholesterol and its derivatives induce the dephosphorylation of BmRpd3, the homolog of mammalian HDAC1, and consequently promote autophagy in *Bombyx mori* [8].

There are 13 HATs identified in mammals, which are classified into three main types including the P300, GCN5, and MYST19 family [11,18]. P300 acetylates Atg5, Atg7, LC3/Atg8, and Atg12, thus inhibit autophagy [10]. Tat-interactive protein 60 kDa (Tip60), the key histone acetyltransferase of the MYST family, binds with acetyl-CoA and subsequently catalyzes the acetylation of proteins, which are involved in the cell cycle, apoptosis, autophagy, and chromatin remodeling [19,20,21]. Tip60 functions as a part of an evolutionarily conserved nuclear multimeric protein complex (NuA4) to acetylate cell cycle-related protein p21 at lysine (lys) 161 and lys163; Tip60 also acetylates the DNA-binding domain at lys120 of p53, which determines the cell fate under DNA damage [22,23]. In addition, the mutation of the Tip60 acetylation site at lys120 in p53 totally abolishes cell apoptosis [24,25]. Tip60 is also implicated in autophagy, Tip60-mediated acetylation of lys162 and lys606 in ULK1 and lys483, lys523, lys533, lys573, and lys633 of Pacer are indispensable for the induction of autophagy in mammals [26,27]. The function of HATs is less studied in insects, and several reports suggest that P300/CBP regulates circadian rhythm in insects [28,29]. However, the function of HATs in autophagy has not been thoroughly investigated.

Nutrients are the most universal upstream signal in regulating autophagy [1]. In mice, cells in most tissues display increased numbers of autophagosomes after starvation [30]. Starvation withdraws growth factors such as insulin and insulin-like growth factors to inhibit MTOR (mechanistic target of rapamycin kinase) signaling, and consequently activates autophagy [31,32]. In addition, starvation also activates AMP-activated protein kinase (AMPK) to induce autophagy through the inhibition of MTOR activity via the phosphorylation of tuberous sclerosis complex 2 (TSC2) and raptor in mammals [32,33]. Similarly, starvation regulates autophagy through interaction with PI3K-MTORC1 and AMPK in *D. melanogaster* and *B. mori*, thus affecting the phosphorylation status of the Atg1-Atg13 protein complex to initiate autophagy [5,34,35].

*B. mori*, a model of lepidopterans, exhibits robust autophagy during larval–pupal metamorphosis induced by high titer 20E combined with starvation signaling [5,7,36]. However, the PTMs in regulating autophagy in insects are not fully elucidated. As a potential acetyltransferase, BmTip60 has not been investigated. Thus, research on its molecular function and regulatory mechanism will provide a better understanding of the regulatory mechanism of multiple biological processes in insects.

## 2. Results

### 2.1. BmTip60 Is Highly Conserved from Insects to Mammals and Is Able to Catalyze Protein Acetylation

**Figure 1:**
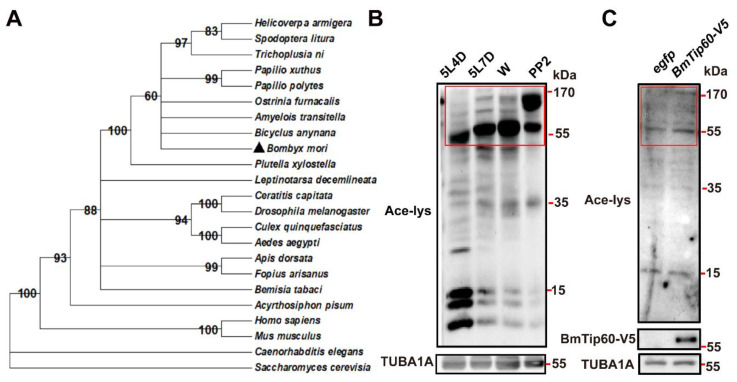
Phylogenetic analysis of the Tip60 homologs and functional identification of BmTip60. (**A**) The phylogenetic tree of Tip60 homologs constructed between 23 species, numbers indicate bootstrap value. (**B**) Acetylation levels of total proteins in fat body at day 4 of 5 larval instar (5L4D), day 7 of 5 larval instar (5L7D), Wandering (W), and PP2 (day 2 of prepupa) stages. Red box: proteins with molecular weight greater than 55 kDa. (**C**) Acetylation levels of total proteins at 48 h after *BmTip60-V5* overexpression in BmN cells. Red box: proteins with molecular weight greater than 55 kDa.

Based on the conserved acetyltransferase domain of mammalian Kat5/Tip60, one predicted *Bombyx* Tip60 (XP_004928298.1) was blasted in NCBI. The phylogenetic tree of Tip60 homologs was constructed between more than 20 species from *Saccharomyces cerevisiae* to *Homo sapiens*, mainly including Insecta, Decapoda, and other Crustacea using the maximum-likelihood approach (gene IDs are listed in the Table S1). Results showed that BmTip60 was highly conserved with the homologs from *Bicyclus anynana* and other lepidopterans, meanwhile, it harbored around 60% identity to the human Kat5/Tip60 (Figure 1A).

Using the antibody against pan-acetylation, the acetylation levels of total proteins were detected in the *B. mori* fat body at day 4 of 5 larval instar (5L4D, middle of the last feeding larval stage), day 7 of 5 larval instar (5L7D, the last day of feeding larval stage), Wandering (W, spinning), and PP2 (day 2 of prepupa) stages. Western blots showed that the acetylated proteins with molecular weights greater than 55 kDa gradually increased while these proteins with molecular weights less than 35 kDa decreased from feeding larval stage to prepupa, showing the diverse variation of acetylation of different proteins simultaneously (Figure 1B). As a putative acetyltransferase, BmTip60 was further detected for the activity of catalyzing the acetylation of proteins. After *BmTip60* overexpression for 48 h, the acetylation levels of proteins with molecular weight greater than 55 kDa were increased in the total proteins of *B. mori* BmN cells, which was consistent with the promotion of protein acetylation at larval–pupal metamorphosis, suggesting the involvement of BmTip60 activity in acetylating these proteins (Figure 1C). Taken together, BmTip60 is a functional acetyltransferase that catalyzes protein acetylation in *B. mori*.

### 2.2. Tissue Distribution of BmTip60 in B. mori 

During larval–pupal metamorphosis, several larval tissues undergo intensive histolysis and programmed cell death [5,37]. In order to reveal the function of BmTip60, its tissue distribution was firstly investigated by bioinformatic analysis in silkworm database 3.0 (https://silkdb.bioinfotoolkits.net, Southwest University, Chongqing, China) [38]. The expression values of *BmTip60* in 17 tissues at different developmental stages (including feeding larvae, wandering, prepupa, pupa and moth) and the subcellular localization of BmTip60 were predicted using ngLOC (an n-gram-based Bayesian classifier), which predicts the subcellular localization of proteins both in prokaryotes and eukaryotes with a prediction accuracy of 89.8% to 91.4% [39]. Results showed that BmTip60 was constitutively expressed in larval fat body from the fourth instar to newly eclosed (day 1) moths, and was especially high at day 3 of the 5 larval instar (5L3D) and in adults; notably, it also exhibited high expression levels in the hemocytes (Supplementary Figure S1A). As predicated, the BmTip60 protein is mainly distributed in the nucleus rather than in the cytoplasm (Supplementary Figure S1B). Moreover, a 3D model displayed by molecule viewer showed that BmTip60’s molecular structure contained a series of α-helix and β-sheet structures (Supplementary Figure S1C). 

**Figure 2:**
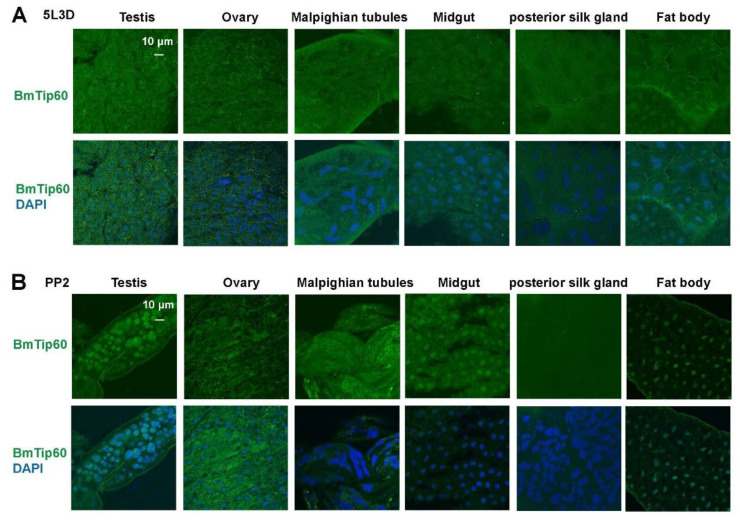
Tissue distribution of immunofluorescent BmTip60 in *B. mori*. (**A**,**B**) Immunofluorescent staining of BmTip60 in testis, ovaries, Malpighian tubules, midgut, posterior silk gland, and fat body at 5L3D and PP2 stages. Green: BmTip60; blue: cell nucleus. Scale bar: 10 micron.

The tissue expression of BmTip60 was detected by immunofluorescent staining in testis, ovaries, Malpighian tubules, midgut, posterior silk gland, and fat body at both 5L3D and PP2 stages. Notably, BmTip60 was distributed evenly in the larval tissue cells, except for a higher signal in some cell nuclei of the midgut and fat body at 5L3D (Figure 2A). In comparison, there was apparently nuclear localization of BmTip60 in the testis, midgut, and fat body at the PP2 stage, indicating the potential roles of BmTip60 in these tissues during larval–pupal transition (Figure 2B). 

### 2.3. Migration of BmTip60 Protein Band Is Consistent with Autophagy Occurrence in Fat Body

**Figure 3:**
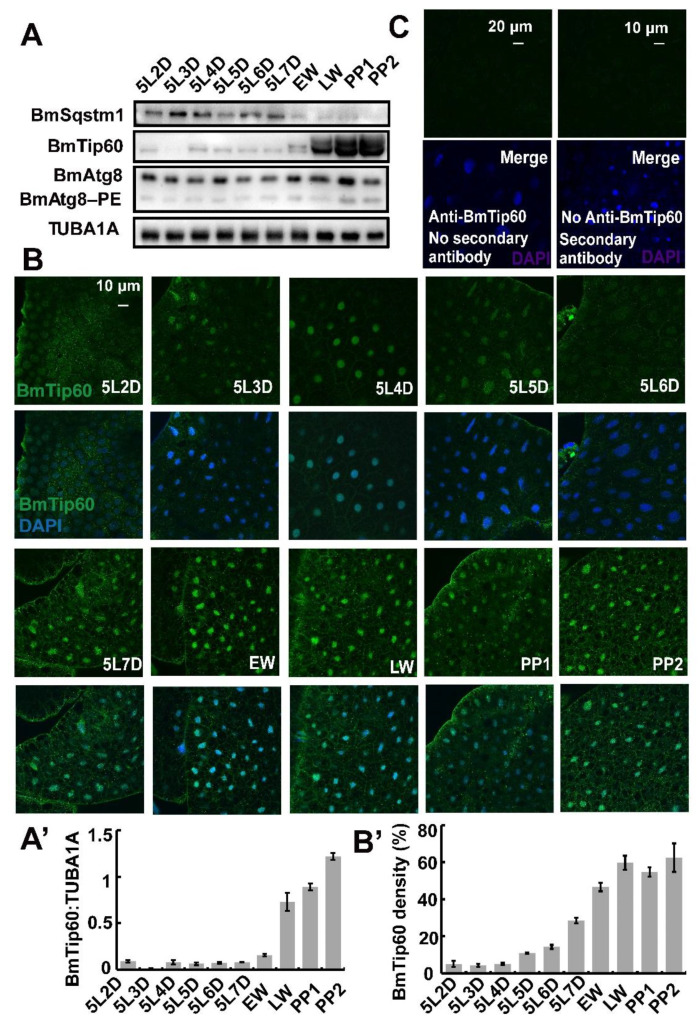
Developmental profiles of BmTip60 protein levels and immunofluorescent staining in the fat body. (**A**) Protein levels of BmSqstm1, BmTip60, and BmAtg8/BmAtg8–PE from 5L2D to PP2. LW: late wandering stage; PP1: day 1 of prepupa. (**A’**) Quantification of total BmTip60 immunoblots in A. (**B**) Immunofluorescent staining of BmTip60 from 5L2D to PP2. Scale bar: 10 micron. (**B’**) Quantification of BmTip60 density in B. (**C**) Negative controls of BmTip60 immunofluorescent staining: fat body immunostained without BmTip60 primary antibody or without fluorescent second antibody. Scale bars: 10 and 20 micron, respectively.

During larval–pupal metamorphosis, autophagy is intensely induced in *B. mori* fat body [5,6,7]. To elucidate the molecular functions of BmTip60 in autophagy, its protein levels were detected in *B. mori* fat body from day 2 of the 5 larval instar (5L2D) to PP2, covering the last larval feeding stage and larval–pupal transition. Western blots showed that BmTip60 (original band, 53 kDa) was significantly increased along with an upward migration of protein band (molecular weight near 57 kDa) from the early wandering (EW) to prepupal stage, implying a modified form of BmTip60 (Figure 3A,A’). Simultaneously, BmAtg8–PE (phosphatidylethanolamine) conjugation and BmSqstm1 degradation were both promoted, indicating the correlation of autophagy occurrence with BmTip60 protein migration (Figure 3A). Moreover, immunofluorescent staining revealed that BmTip60 protein density increased in the fat body at late larval stages (Figure 3B,C). Overall, BmTip60 protein is putatively modified and increased in both nuclei and cytoplasm during larval–pupal metamorphosis when autophagy occurs. 

### 2.4. Starvation but Not 20E Promotes BmTip60 in Addition to Autophagy Induction in B. mori

**Figure 4:**
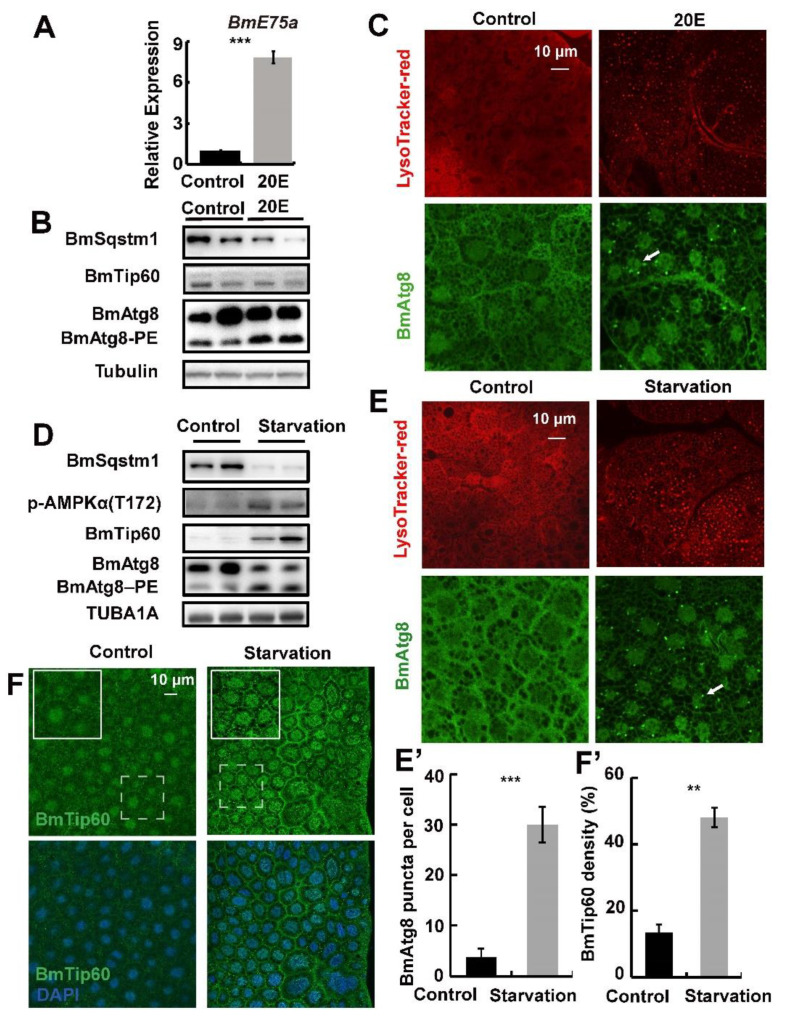
Starvation but not 20-hydroxyecdysone (20E) promotes BmTip60 in addition to autophagy in *B. mori* fat body. (**A**) mRNA levels of *BmE75a* after 20E treatment for 24 h. (**B**) Protein levels of BmSqstm1, BmTip60, and BmAtg8 after 20E treatment, two repeats are shown here. (**C**) LysoTracker red staining and BmAtg8 immunofluorescent staining after 20E treatment. Arrow: BmAtg8 puncta. Scale bar: 10 micron. (**D**) Protein levels of BmSqstm1, BmTip60, p-AMPKα (T172), and BmAtg8 after starvation for 24 h. Two repeats are shown here. (**E**) LysoTracker red staining and BmAtg8 immunofluorescent staining after starvation. Arrow: Atg8 puncta in the cytoplasm. Scale bar: 10 micron. (**E’**) Quantification of BmAtg8 puncta in E. (**F**) Immunofluorescent staining of BmTip60 after starvation. White solid box: magnified field. White dashed box: original field. Scale bar: 10 micron. (**F’**) Quantification of BmTip60 density in F. ** *p* < 0.01; *** *p* < 0.001.

Starvation is the universal regulator of autophagy in eukaryotes, and it induces autophagy by inhibiting MTOR signaling coordinated with 20E signaling in fat body during larval–pupal metamorphosis in *B. mori* [5,8,36]. In order to illustrate the regulation of BmTip60 by starvation and 20E, 5L3D larvae were injected with 10 μg/larva 20E or treated with food deprivation for 24 h, and the fat body was collected for further analysis. *BmE75a*, a 20E primary response gene, was upregulated for more than five folds in the fat body after 20E injection (Figure 4A). Subsequently, monitored by BmAtg8–PE conjugation, BmSqstm1 degradation, and lysosome acidification staining by LysoTracker red autophagy was induced along with increased BmAtg8 puncta in the cytoplasm of fat body cells after 20E treatment, whereas, the protein levels of BmTip60 were not significantly affected (Figure 4B,C). In comparison, starvation upregulated the protein levels of both BmTip60 and p-AMPKα, which was changed in accordance with starvation-induced autophagy (Figure 4D,E’). Accordingly, BmTip60 immunofluorescent staining indicated that its intensity increased by nearly 3-fold when induced by starvation in *B. mori* fat body (Figure 4F,F’).

### 2.5. AMPK Activity Mediates the Migration of BmTip60 Protein 

**Figure 5:**
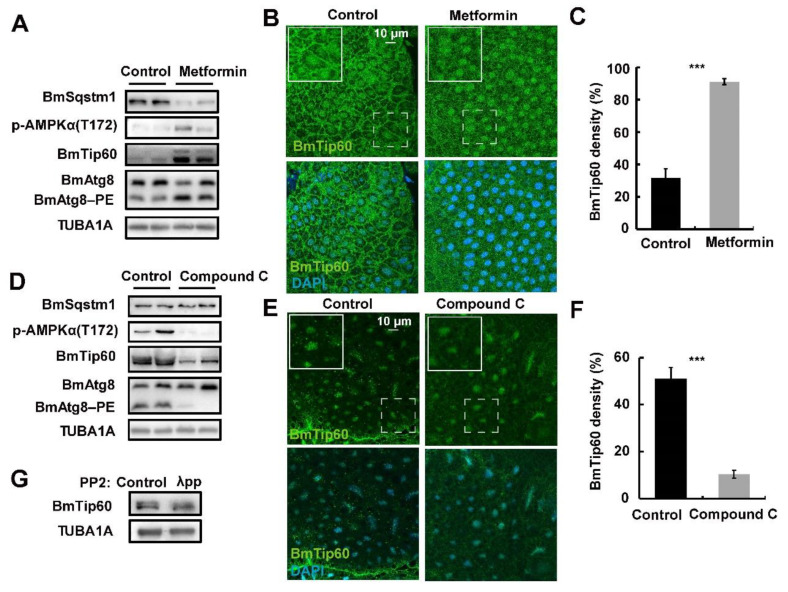
AMP-activated protein kinase (AMPK) activity and phosphorylation mediate the migration of BmTip60 upper band in *B. mori* fat body. (**A**) Protein levels of BmSqstm1, BmTip60, p-AMPKα (T172), and BmAtg8 after metformin treatment for 24 h, two repeats are shown here. (**B**) Immunofluorescent staining of BmTip60 in *B. mori* after metformin treatment for 24 h. Scale bar: 10 micron. White solid box: magnified field. White dashed box: original field. (**C**) Quantification of BmTip60 density in B. (**D**) Protein levels of BmSqstm1, BmTip60, p-AMPKα (T172), and BmAtg8 after compound C treatment for 24 h, two repeats are shown here. (**E**) Immunofluorescent staining of BmTip60 after compound C treatment for 24 h. White solid box: magnified field. White dashed box: original field. (**F**) Quantification of BmTip60 density in E. (**G**) Protein level of BmTip60 after λ-phosphatase treatment. *** *p* < 0.001.

Starvation activates AMPK (AMP-activated protein kinase) in addition to the inhibition of MTOR signaling in insects, and AMPK antagonizes or coordinates with MTOR signals in regulating the phosphorylation of different proteins [5,33,34,35]. BmTip60 harbored an additional protein band in *B. mori* fat body during the larval–pupal metamorphosis (Figure 3A), when AMPK activity is high [33]. Thus, we questioned whether AMPK activity was involved in regulating the migration of BmTip60 proteins. Metformin, the AMPK-specific activator, was injected into 5L3D larvae when AMPK activity is low [33,40]. After metformin treatment for 24 h, fat body was collected for Western blots and immunofluorescent staining. Results showed that the protein levels of p-AMPKα were promoted; significantly, the upper band of BmTip60 was induced in addition to the promotion of the original band after metformin treatment. Meanwhile, BmAtg8–PE conjugation and BmSqstm1 degradation were increased, indicating the induction of autophagy after AMPK activation (Figure 5A). Moreover, immunofluorescent staining indicated that BmTip60 density was notably increased after metformin treatment (Figure 5B,C). In contrast, compound C, the AMPK-specific inhibitor, was injected into the larvae at initiation of wandering (IW) stage, when AMPK activity is relatively high [33,40]. Western blots showed that protein levels of the BmTip60 upper band and p-AMPKα were robustly reduced, along with a decrease in BmAtg8-PE conjugation and accumulation of BmSqstm1 (Figure 5D). Immunofluorescent staining indicated that compound C treatment reduced BmTip60 protein (Figure 5F,G). As AMKP regulates the phosphorylation modification of proteins, thus we hypothesized that the upper protein band of BmTip60 was a phosphorylated form. After treatment with λ-phosphatase, the upper band of BmTip60 was partly abolished, indicating that it was an authentic phosphorylated form of BmTip60 (Figure 5D). Taken together, the AMPK signal positively regulates the phosphorylation of BmTip60, leading to the migration of its protein in *B. mori*. 

### 2.6. Ser99, an Evolutionarily Conserved Phosphorylation Site, Is Indispensable for BmTip60-Mediated Autophagy 

**Figure 6:**
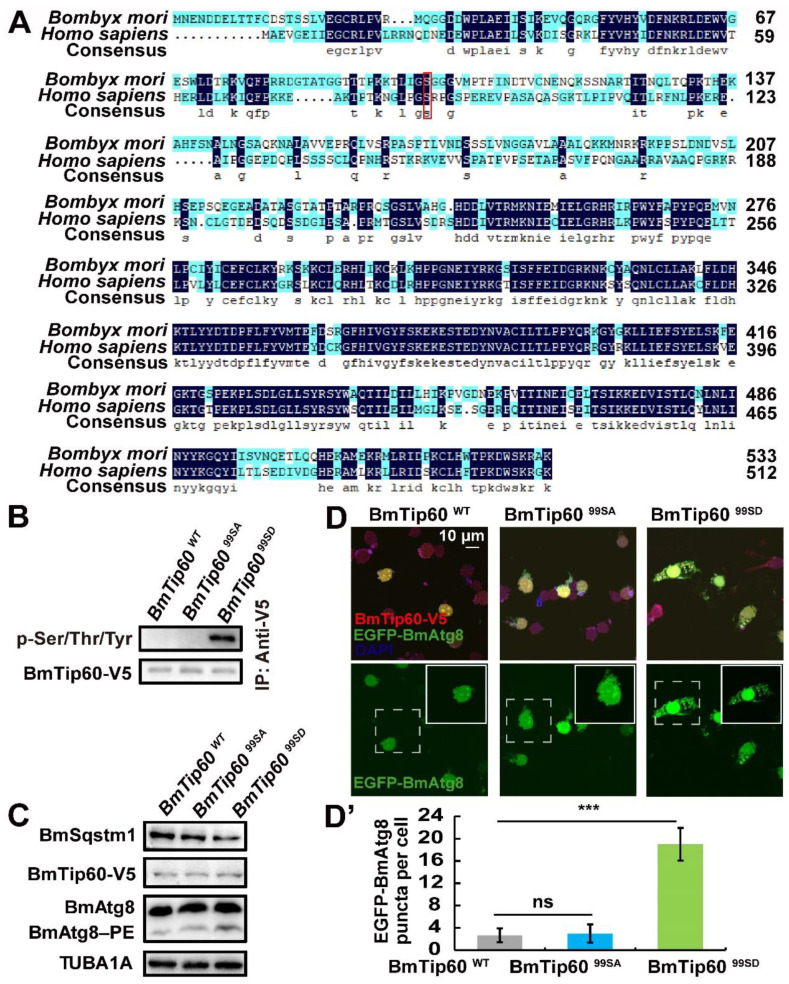
Phosphorylation at Ser99 is required for BmTip60-mediated autophagy. (**A**) Amino acid sequences of Tip60 homologs in *B. mori* and *H. sapiens* are aligned by DNAMAN software. Dark blue: conserved amino acid sequence, light blue: nonconservative amino acid sequence. Red box: the conserved phosphorylation site. (**B**) Phosphorylation level of immunoprecipitated BmTip60 after overexpression of *BmTip60-V5*, *BmTip60-99SA-V5*, and *BmTip60-99SD-V5* in BmN cells for 48 h, respectively. WT: wild-type; 99SA: Serine99 mutated to Alanine99; 99SD: Serine99 mutated to Aspartic99. (**C**) Protein levels of BmSqstm1, BmTip60, and BmAtg8 after overexpression of *BmTip60-V5*, *BmTip60-99SA-V5* or *BmTip60-99SD-V5* in BmN cells for 48 h. (**D**) Observation of EGFP-BmAtg8 and immunostained BmTip60-V5/BmTip60-99SA-V5/BmTip60-99SD-V5 under nutrient-rich conditions in BmN cells. Scale bar: 10 micron. White solid box: magnified field. White dashed box: original field. (**D’**) Quantification of EGFP-BmAtg8 puncta in (D). *** *p* < 0.001.

In mice, the phosphorylation of Tip60 at Ser86 under starvation leads to its activation, and subsequently, the acetylation of ULK1 and autophagy induction [26]. Thus, amino acid sequences *H. sapiens* Tip60 and BmTip60 were firstly aligned using DANMAN8.0 software. Alignment showed that one phosphorylation site, which is conserved with the human homolog, was predicated in BmTip60 at Ser99 (Figure 6A). To verify the authenticity of Ser99 phosphorylation, two *BmTip60* mutants were constructed, including the inactive mutant *BmTip60^99SA^* (Serine99 mutated to Alanine99) and the constitutive-activated mutant *BmTip60^99SD^* (Serine99 mutated to Aspartic99). After overexpression in BmN cells for 48 h, BmTip60-V5, BmTip60^99SA^-V5, and BmTip60^99SD^-V5 were immunoprecipitated and detected by Western blots using an antibody against pan-phosphoserine/threonine/tyrosine-phosphorylation. Results showed that only overexpressed BmTip60^99SD^ was detected with immunoblots (Figure 6B). Moreover, *BmTip60^99SD^* overexpression caused premature autophagy as indicated by BmSqstm1 degradation and BmAtg8–PE conjugation under nutrient-rich condition compared to *BmTip60* and *BmTip60^99SA^* overexpression in BmN cells (Figure 6C). Importantly, only *BmTip60-99SD* and *egfp-BmAtg8* co-overexpression induced significant EGFP-BmAtg8 punctation under nutrient-rich conditions (Figure 6D,D’). These results indicate that the phosphorylation of BmTip60 at Ser99 is conserved with the human homolog Tip60 and is indispensable for BmTip60-mediated autophagy in *B. mori*.

## 3. Discussion

### 3.1. Phosphorylation of BmTip60 Is Regulated by AMPK Activity and Requisite for Autophagy Induction

In mice and humans, acetyltransferase Tip60 harbors two conserved phosphorylation sites at Ser86 and Ser90 [26]. Phosphorylation of Tip60 at Ser86 is a potential for serum starvation-induced autophagy through the acetylation of ULK1 and consequent initiation of autophagosome formation. In addition, Tip60 acetylates Pacer under nutrient deprivation conditions, which is further able to cooperate with Stx17 in the recruitment of the HOPS (homotypic fusion and protein sorting) complex to autophagosomes [20,26,27]. Similarly, Esa1p, the yeast homolog of mammalian Tip60, catalyzes the hyperacetylation of Atg3, which enhances its interaction with Atg8 and facilitates Atg8 lipidation as well as autophagosome maturation under starvation conditions [41]. Protein acetylation and deacetylation are suggested in diapause [15], circadian rhythm [28,29], stress resistance and innate immunity in insects [14,15]. In *Drosophila*, the Tip60 protein complex promotes germline stem cell daughter differentiation in females [42], whereas, the regulatory mechanisms of Tip60 homologs in autophagy are not well documented in insects. 

Here, we functionally identify BmTip60 in *B. mori* and reveal a part of the regulatory mechanism of BmTip60-mediated autophagy. BmTip60 harbors a high identity to the lepidopterans and mammalian homologs and is able to catalyze the acetylation of a series of proteins in *B. mori* (Figure 1). Western blots show that BmTip60 protein is migrated to form a greater band from the EW to prepupal stages in fat body, when autophagy is intensively induced (Figure 2 and Figure 3). In mammals, Tip60 is phosphorylated when autophagy is triggered by starvation. Serum deprivation deinhibits glycogen synthase kinase-3 (GSK3), which promotes the phosphorylation of BmTip60 at Ser86, and subsequent activation of autophagy through the acetylation of ULK1 [26], whereas autophagy is as effectively induced by serum starvation in AMPKα1/2-DKO (double knockout) mouse embryonic fibroblast (MEF) cells as in control cells, indicating the independence of AMPK in autophagy induction in this case [26,27]. In *B. mori*, comparisons between the variations of BmTip60 proteins, autophagy occurrence, and nutrient signals including p-InR, p-4EBP, and p-AMPK from 5L2D to PP2 in fat body suggested that AMPK is the most possible protein kinase to phosphorylate BmTip60 at the late larval and prepupal stages [5,33,40]. As expected, metformin and compound C treatments, which activate and inhibit AMPK activity, result in the induction and abolishment of BmTip60 protein migration. Subsequently, λ-phosphatase treatment confirms that the migrated band is a phosphorylated form of BmTip60. In summary, AMPK activity mediates the phosphorylation of BmTip60 at the prepupal stage (Figure 5).

In order to investigate the function of BmTip60, one conserved phosphorylation site at Ser99 is predicated by alignment with the homolog of *H. sapiens* Tip60. Similar to the mammalian homolog, the phosphorylation of BmTip60 at Ser is requisite for autophagy promotion (Figure 6). Interestingly, Ser99 is conserved in the homologs of lepidopterans but not *D. melanogaster*, implying that the functions of phosphorylation at Ser86/Ser99 are conserved in lepidopterans and mammals while maybe not in other insects. Besides, phosphorylation site Ser90 in mammalian Tip60, which may be a prerequisite for the phosphorylation of Ser86, is absent in BmTip60, but the great molecular weight of phosphorylated BmTip60 suggests that there may exist other phosphorylation sites in BmTip60. In general, the precise regulatory mechanisms of Tip60 in insects are waiting for further investigation, which will help us to fully understand the molecular functions of HATs.

### 3.2. 20E Functions Differently with Starvation in Regulating BmTip60

Starvation and 20E are the two major stimuli of autophagy in insects [5,7,35,43]. In *B. mori* and *D. melanogaster*, 20E-EcR/USP signaling predominantly upregulates autophagy through the induction of *Atg* gene expression in addition to antagonizing insulin/insulin-like growth factor (IGF) signaling (IIS) [5,33]. The 20E primary-response genes such as *E75* and *E93* are pivotal for 20E-induced autophagy during larval–pupal metamorphosis [6,44]. 20E and starvation treatments both induce the gene expressions of *B. mori V-ATPases* subunits and their assembly through the activation of transcription factor *TFEB* and inhibition of MTOR activity, resulting in lysosome acidification and autophagic flux [36]. In addition, 20E induces starvation-like conditions by reducing food consumption [5,33,45]. In general, 20E interacts closely with starvation signals, and they function similarly to each other in regulating autophagosome formation as well as autophagic flux in most cases. However, in this study we find that BmTip60 protein is only promoted after starvation, but not 20E treatment, suggesting that they function differently in inducing autophagy at least partly through regulating BmTip60 (Figure 4). Thus, how BmTip60 is differently regulated by starvation and 20E is worthy of further investigation.

## 4. Materials and Methods

### 4.1. Silkworms and Cells

The silkworm larvae (Dazao), provided by the Sericultural and Agri-Food Research institute of the Guangdong Academy of Agricultural Sciences (Guangzhou, China), were fed on fresh mulberry leaves in the laboratory at 25 °C under a 14 h light/10 h dark cycle [5,6,8]. *B. mori* BmN cells were maintained in Grace’s insect medium (Sigma-Aldrich, Darmstadt, Germany, G9771) supplemented with 10% heat-inactivated fetal bovine serum (Gibco, 10100-147).

### 4.2. Plasmid Construction and Transfection

Total RNA was isolated from cells using Trizol (Vazyme, Nanjing, China, R401-01). Reverse transcription was performed using M-MLV reverse transcription reagents (Takara, Dalian, China, 639522). Full-length *BmTip60* was cloned from total cDNA obtained from prepupal *B. mori* fat body and fused with V5 tag by PCR (using primers BmTip60-F and BmTip60-R), and inserted into pIEx4 overexpression vector via Nco I (NEB, Ipswich, Massachusetts, USA, R3193) and Kpn I (NEB, Ipswich, Massachusetts, USA, R3142) restriction enzyme sites. pIEx-4-egfp-BmAtg8 was previously constructed [8]. *BmTip60* and *egfp-BmAtg8* plasmids were transfected into BmN cells using FuGENE^®®^ HD Transfection Reagent (Promega, Madison, Wisconsin, UAS, E2311) according to the manufacturer’s instructions. After plating in the 12-well plates per well (Guangzhou Jet Bio-Filtration Co., Ltd., Guangzhou, China, TCP-010-006) for 24 h, the BmN cells were transfected with 1 μg DNA of plasmid for 48 h, and then subjected to further assays. All primers used in this paper are listed in Table S2. 

### 4.3. Starvation and 20E Treatment

The *B. mori* larvae from day 3 of 5 instar (5L3D) were treated with starvation or 20E injection (Sigma-Aldrich, Darmstadt, Germany, H5142; 10 µg/larva) for 24 h. For the 20E injection, 5 µL of the solution was injected into hemolymphs through the second pleopod of *B. mori*, and the control larvae were injected with the same volume of solvent according to the previously described methods [7,36]. Ten animals were used in each group and three biological replicates were conducted. 

### 4.4. Chemical Treatments

Metformin (Selleck, Houston, Texas, USA, S1950; 30 μg/larva) and compound C (Sigma-Aldrich, P5499; 5 μg/larva) was injected into *B. mori* larvae at 5L3D or 12 h before IW, respectively. The control larvae were injected with the same volume of solvent (5 μL). After treatment for 24 h, the fat body was collected for further analysis. Ten animals were included in each group and three biological replicates were performed.

### 4.5. Immunoprecipitation and Western Blots

BmN cells overexpressing *BmTip60*, *BmTip60^99SD^*, or *BmTip60^99SA^* for 48 h were harvested and lysed in Nonidet P-40 lysis buffer (Beyotime Biotechnology Co., Ltd., Shanghai, China, P0013F) supplemented with protease inhibitor cocktail (ThermoFisher Scientific, Waltham, Massachusetts, USA, 78429; 1:100). The supernatant of lysed cells was preincubated with a V5 antibody (Abcam, Cambridge, UK, ab27671; 1:200, *v*/*v*) at 4 °C for 2 h. Then, the mixtures were further incubated with agarose beads (Thermo Fisher Scientific, Waltham, Massachusetts, USA, 20421) at 4 °C overnight according to a standard immunoprecipitation procedure.

Fat body and BmN cells were lysed in RIPA lysis buffer (Beyotime Biotechnology Co., Ltd., Shanghai, China, P0013B) supplied with protease inhibitor phenyl methane sulfonyl fluoride (PMSF) for 30 min on ice, and protein concentrations were determined by the BCA protein assay kit (Beyotime Biotechnology Co., Ltd., Shanghai, China, P0012). Total proteins in samples were separated by SDS-PAGE gels and subsequently transferred to the polyvinylidene difluoride (PVDF) membrane followed by the standard procedure of Western blots. Immunoblotting was performed using the primary antibodies against BmTip60 (Bioss, Boston, Massachusetts, USA, bs-13686R; 1:1000), V5 (Abcam, Cambridge, UK, ab27671; 1:2000), or pan-phosphoserine/threonine/tyrosine-phosphorylation (Abcam, Cambridge, UK, ab15556; 1:2000) overnight. Tubulin was used as the reference protein (Beyotime Biotechnology Co., Ltd., Shanghai, China, AT819; 1:5000) [37]. Images were visualized using Tanon-5200 imager. Quantification of immunoblots were performed by gray scanning using Image J [46]. 

### 4.6. Immunofluorescent Staining

BmN cells and fat body were fixed in 4% paraformaldehyde, respectively, for 0.5 h and 1 h followed by PBS washing for three times. The cells or tissues were permeabilized with 1% triton X-100 (Beyotime Biotechnology Co., Ltd., Shanghai, China, P0096) for 1 h at room temperature, and then blocked with 5% BSA for 2 h at 4 °C followed by incubation with the primary antibodies (dilution at 1:200) at 4 °C overnight. The cells and fat body were further incubated with an Alexa Fluor-488-conjugated secondary antibody (Thermo Fisher Scientific, Waltham, Massachusetts, USA, A11008; 1:200) for 2 h at room temperature. The nucleus was stained with DAPI (Beyotime Biotechnology Co., Ltd., Shanghai, China, C1006; 1:1000) for 30 min at room temperature. Images were taken under an Olympus FV300 microscope (Olympus, Tokyo, Japan) [6,8]. Immunofluorescent BmTip60 in 150–200 fat body cells from three independent biological repeats was quantified by its density compared to the whole density of the calculated area in the images, and punctation of BmAtg8 was also recorded and analyzed in a total of 50–200 cells using Image J.

### 4.7. LysoTracker Staining

Fat body from *B. mori* was stained with LysoTracker Red DND-99 (Thermo Fisher Scientific, Waltham, Massachusetts, USA, L7528) in PBS (pH 7.0) at a final concentration of 50 nM for 10 min at 37 °C and subsequently washed with PBS three times. Observations were performed under an FV3000 confocal microscope. 

### 4.8. λ-Phosphatase Treatment of B. mori Fat Body Cells

Fat body from PP2 stages was collected and extracted for total proteins in RIPA lysis buffer, which were further treated with λ-phosphatase (Sigma-Aldrich, Darmstadt, Germany, P9614) according to the manufacturer’s instructions. After treatment, the total proteins were subjected to Western blots analysis detected by BmTip60 antibody.

### 4.9. Mutation of Ser99 in BmTip60

*BmTip60*-point mutation including pIEx4-Tip60^99SA^ and pIEx4-Tip60^99SD^ was performed by PCR using a pIEx4-Tip60^WT^ vector as a template catalyzed by Taq DNA polymerase (TaKaRa, Dalian, China, R10T1) (primers for Ser99 to Asp99 mutation, F: 5′-CACTCATTGGAGACGGAGGTGGCGT-3′, R: 5′-ACCTCCGTCTCCAATGAGTGTTTTC-3′; primers for Ser99 to Ala99 mutation, F: 5′-CACTCATTGGAGCAGGAGGTGGCGT-3′, R: 5′-ACCTCCTGCTCCAATGAGTGTTTTC-3′), respectively. The PCR products was digested by DpnI (NEB, Ipswich, Massachusetts, USA, R0176) restriction enzyme to degrade the redundant templates and further subjected to molecular cloning procedure.

### 4.10. Statistical Analysis

The experimental data were analyzed by a Student’s *t*-test, * *p* < 0.05; ** *p* < 0.01; *** *p* < 0.001. 

## 5. Conclusions

This study identifies the molecular function and regulation of BmTip60 in response to nutrient deficiency. We demonstrate that starvation induces the protein promotion of BmTip60 by activating AMPK signals. In addition, Ser99, which is conserved in lepidopterans and mammals, is identified as a functional phosphorylation site for BmTip60-mediated autophagy. This study provides a foundation for further studies on BmTip60 and other HATs in the future.

## Supplementary Materials

Supplementary materials can be found at https://www.mdpi.com/1422-0067/21/18/6893/s1. Figure S1: Bioinformatics analysis of BmTip60 in silkdatabase3.0. Table S1: All GeneID used in phylogenetic tree analysis. Table S2: The primers used in this study.

## Abbreviations

20E	20-hydroxyecdysone
Tip60	Tat-interactive protein, 60 KDa
*Atg*	Autophagy-related
AMPK	AMP-activated protein kinase
*B. mori*	*Bombyx mori*
BmSqstm1	*Bombyx* sequestosome 1
lys	Lysine
MTOR	mechanistic target of rapamycin kinase
PBS	Phosphate buffered saline
PTMs	Post-translational modifications
HATs	Histone acetyltransferases
