# Correction: Wu, W., et al. Tip60 Phosphorylation at Ser 99 Is Essential for Autophagy Induction in *Bombyx mori*. *Int. J. Mol. Sci*. 2020, *21*, 6893

**DOI:** 10.3390/ijms22041751

**Published:** 2021-02-10

**Authors:** Wenmei Wu, Kang Li, Haigang Zhao, Xianying Xu, Jing Xu, Man Luo, Yang Xiao, Ling Tian

**Affiliations:** 1 Guangdong Provincial Key Laboratory of Agro-Animal Genomics and Molecular Breeding/Guangdong Provincial Sericulture and Mulberry Engineering Research Center, College of Animal Science, South China Agricultural University, Guangzhou 510642, China; wuwenmei@stu.scau.edu.cn (W.W.); 20183139062@stu.scau.edu.cn (X.X.); zhibaoxujing@stu.scau.edu.cn (J.X.); luoman@stu.scau.edu.cn (M.L.); 2 Guangdong Province Key Laboratory for Biotechnology Drug Candidates, School of Life Sciences and Biopharmaceutics, Guangdong Pharmaceutical University, Guangzhou 510006, China; 3 Guangdong Provincial Key Laboratory of Insect Developmental Biology and Applied Technology, Institute of Insect Science and Technology, School of Life Sciences, South China Normal University, Guangzhou 510631, China; likang0118@foxmail.com (K.L.); hgzhao@sibs.ac.cn (H.Z.); 4 The Sericultural and Agri-Food Research Institute of the Guangdong Academy of Agricultural Sciences, Guangzhou 510610, China; xiaoyang@gdaas.cn

The author wishes to make the following correction to this paper [1]. Due to an error involving the immunofluorescent staining for BmAtg8 after starvation treatment in Figure 4E, it should be replaced with the correct new figure (Figure 1). Due to a change in the statistical analysis between the three groups as well as the replacements of the merged and magnified pictures for BmTip60^WT^ in Figure 6D, the corrected figure is shown below (Figure 2).

The authors would like to apologize for any inconvenience caused to the readers by these changes.

## Figures and Tables

**Figure 1:**
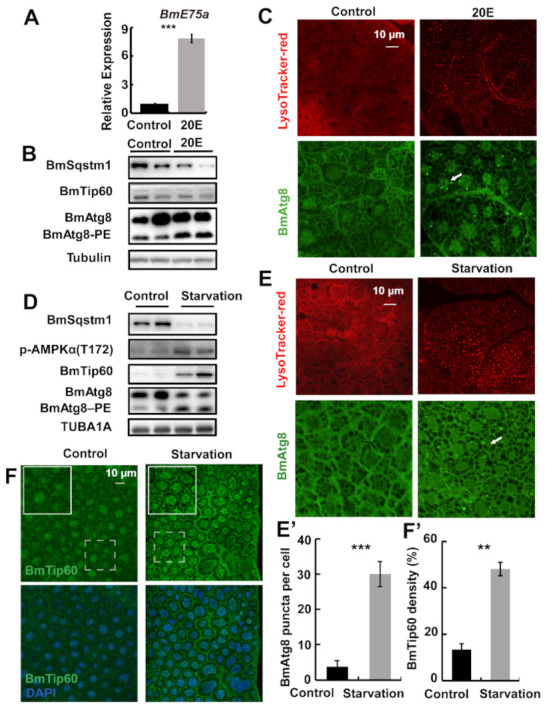
Starvation but not 20-hydroxyecdysone (20E) promotes BmTip60 in addition to autophagy in *B. mori* fat body. (**A**) mRNA levels of *BmE75a* after 20E treatment for 24 h. (**B**) Protein levels of BmSqstm1, BmTip60, and BmAtg8 after 20E treatment. Two repeats are shown here. (**C**) LysoTracker red staining and BmAtg8 immunofluorescent staining after 20E treatment. Arrow: BmAtg8 puncta. Scale bar: 10 micron. (**D**) Protein levels of BmSqstm1, BmTip60, p-AMPKα (T172), and BmAtg8 after starvation for 24 h. Two repeats are shown here. (**E**) LysoTracker red staining and BmAtg8 immunofluorescent staining after starvation. Arrow: Atg8 puncta in the cytoplasm. Scale bar: 10 micron. (**E’**) Quantification of BmAtg8 puncta in (E). (**F**) Immunofluorescent staining of BmTip60 after starvation. White solid box: magnified field. White dashed box: original field. Scale bar: 10 micron. (**F’**) Quantification of BmTip60 density in (**F**). One-way analysis of variance was performed by SPSS in the whole figure, * *p* < 0.05; ** *p* < 0.01; *** *p* < 0.001, n = 200 cells.

**Figure 2:**
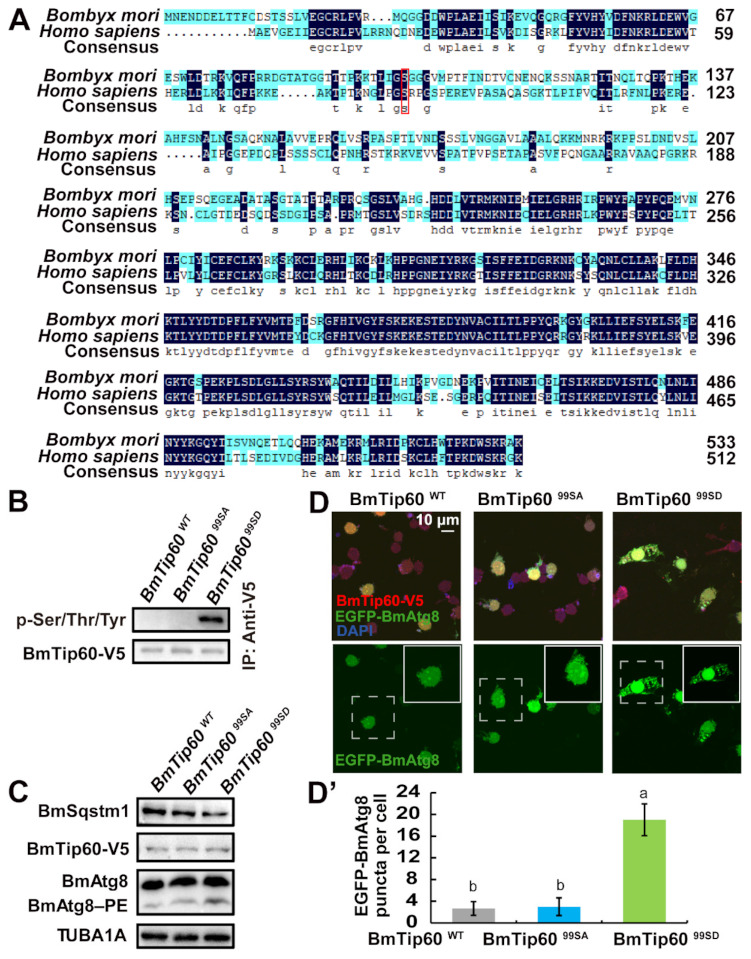
Phosphorylation at Ser99 is required for BmTip60-mediated autophagy. (**A**) Amino acid sequences of Tip60 homologs in *B. mori* and *H. sapiens* are aligned by DNAMAN software. Dark blue: conserved amino acid sequence, light blue: nonconservative amino acid sequence. Red box: the conserved phosphorylation site. (**B**) Phosphorylation level of immunoprecipitated BmTip60 after overexpression of *BmTip60-V5*, *BmTip60^99SA^-V5*, and *BmTip60^99SD^-V5* in BmN cells for 48 h, respectively. WT: wild-type; 99SA: Serine99 mutated to Alanine99; 99SD: Serine99 mutated to Aspartic99. (**C**) Protein levels of BmSqstm1, BmTip60, and BmAtg8 after overexpression of *BmTip60-V5*, *BmTip60^99SA^-V5*, or *BmTip60^99SD^-V5* in BmN cells for 48 h. (**D**) Observation of EGFP-BmAtg8 and immunostained BmTip60-V5/BmTip60^99SA^-V5/BmTip60^99SD^-V5 under nutrient-rich conditions in BmN cells. Scale bar: 10 micron. White solid box: magnified field. White dashed box: original field. (**D’**) Quantification of EGFP-BmAtg8 puncta in (D). One-way analysis of variance was performed by SPSS in the whole figure; different lowercase letters mean *p* < 0.05, n = 50 cells.
