# Supplementary material for: Tip60 Phosphorylation at Ser 99 Is Essential for Autophagy Induction in Bombyx mori
Source: Int J Mol Sci. 2020 Sep 20;21(18):6893. doi: 10.3390/ijms21186893 (PMC7555017; doi:10.3390/ijms21186893)
Supplement: Supplementary file 1 [file ijms-21-06893-s001.pdf]

International Journal of Molecular Sciences

MDPI

Supplementary Materials:

Figure S1: Bioinformatics analysis of *BmTip60* in silkdatabase3.0.

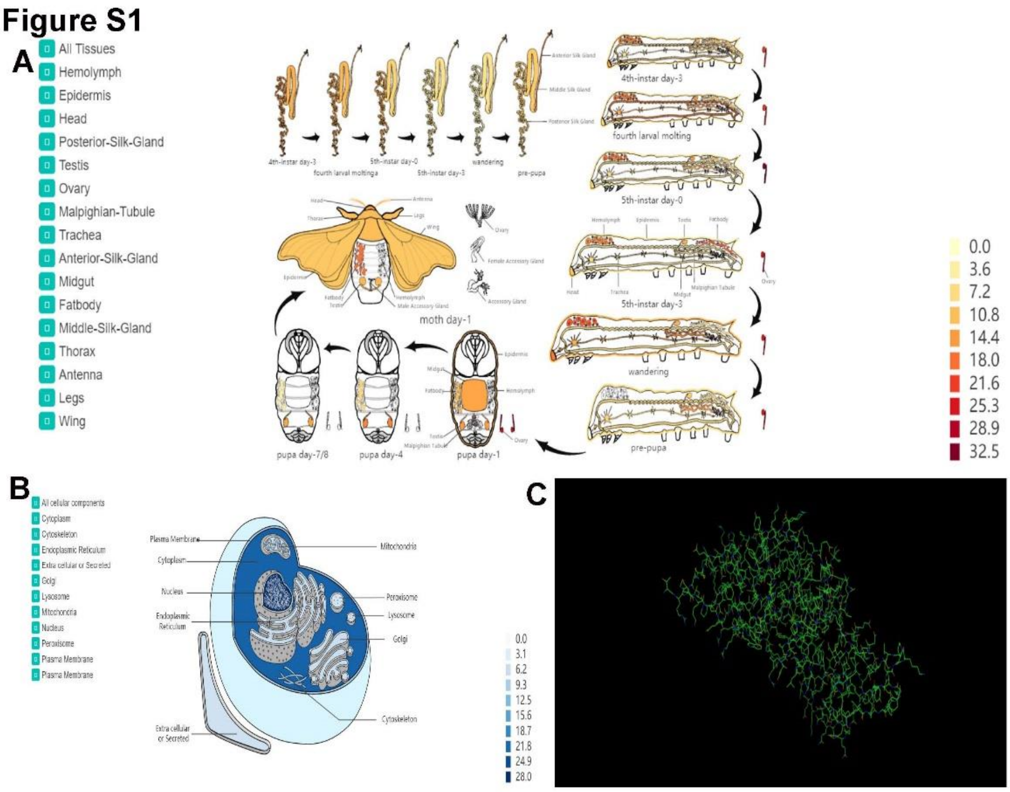

Supplementary Figure S1. Bioinformatics analysis of *BmTip60* in silkdatabase3.0. **(A)** The expression values of *BmTip60* in 17 tissues at different growing stages (instar, larval, wandering, pupa, and moth) is indicated by different colors. Light yellow: expression value is 0, deep red: the highest expression value. **(B)** The predicted subcellular localization of BmTip60. **(C)** 3D model of molecular structure of BmTip60 protein, red color: α-helix, green color: β-sheet, and dark blue color: random coil.

Table S1: *GeneIDs used* in *phylogenetic tree* study

Table S1. *GeneIDs used in phylogenetic tree*

Species	GeneID
Acyrthosiphon pisum	XP_008181995.1
Aedes aegypti	XP_021706851.1
Amyelois transitella	XP_013194528.1
Apis dorsata	XP_006618580.1
Bemisia tabaci	XP_018900801.1
Bicyclus anynana	XP_023947155.1

Bombyx mori	XP_004928298.1
Caenorhabditis elegans	NP_504796.1
Ceratitis capitata	XP_004530447.1
Culex quinquefasciatus	XP_001842875.1
Drosophila melanogaster	NP_001259234.1
Fopius arisanus	XP_011302800.1
Helicoverpa armigera	XP_021201592.1
Homo sapiens	NP_874369.1
Leptinotarsa decemlineata	XP_023025142.1
Mus musculus	NP_848752.1
Ostrinia furnacalis	XP_028156455.1
Papilio polytes	XP_013139927.1
Papilio xuthus	XP_013173093.1
Plutella xylostella	XP_011547865.1
Saccharomyces cerevisia	EDZ69167.1
Spodoptera litura	XP_022826725.1
Trichoplusia ni	XP_026731014.1

Table S2: The primers used in this study

Table S2. Primers used for vector construction

Primer name	Primer sequences[1] (5′ to 3′)
BmTip60-F	CATGCCATGGCAATGAACGAAAATGATGATGAATTGACAACA
BmTip60-R	CGGGGTACCTCACGTAGAATCGAGACCGAGGAGAGGGTTAGGGATAGG
	CTTACCCCATTTCGCCCGTTTAGACCAATCTT
BmAtg8-F	GGAAGATCTGAAATTCCAATACAAAGAAG
BmAtg8-R	CGGGGTACCTTAATTTCCATAGACATTTTCGTC

[1]Sequences in underlined represent enzyme cutting sites. CCATGG indicated Nco I enzyme site, GGTACC indicated Kpn I enzyme site, AGATCT indicated Bgl II enzyme site.
